# Urological Challenges during Pregnancy: Current Status and Future Perspective on Ureteric Stent Encrustation

**DOI:** 10.3390/jcm13133905

**Published:** 2024-07-03

**Authors:** Francesco Di Bello, Gianluigi Califano, Simone Morra, Claudia Collà Ruvolo, Agostino Fraia, Gabriele Pezone, Ernesto Di Mauro, Salvatore Aprea, Luigi Napolitano, Gabriele Saccone, Massimiliano Creta, Nicola Longo

**Affiliations:** Department of Neurosciences, Reproductive Sciences and Odontostomatology, University of Naples “Federico II”, 80131 Naples, Italy; francesco.dibello@unina.it (F.D.B.); gianl.califano2@gmail.com (G.C.); simonemorra@outlook.com (S.M.); agostino.fraia@gmail.com (A.F.); gabrielepezone94@gmail.com (G.P.); ernestodm9@gmail.com (E.D.M.); sa.aprea@gmail.com (S.A.); dr.luiginapolitano@gmail.com (L.N.); gabriele.saccone@unina.it (G.S.); max.creta@gmail.com (M.C.); nicola.longo@unina.it (N.L.)

**Keywords:** forgotten stent, urology, calcified stent, gestational age, stone disease

## Abstract

The management of ureter hydronephrosis and urolithiasis during pregnancy has been changed by the adoption of ureteric stents. Despite their broad use for several other conditions, from emergency to elective settings, their complications cannot be ignored. Being most prevalent during pregnancy, urinary tract infections and stent encrustations are particularly common and can affect either fetal growth or maternal–fetal homeostasis, leading to obstetric complications. The main concern associated with ureteric stents is the indwelling time, which could represent the potential trigger of those complications. However, to ensure the optimal management of a ureteric stent during pregnancy, factors such as the grading of encrustations and the presence, size, and location of stones should be evaluated in pre-operative planning. As a consequence, a multimodal approach, including obstetrics, gynecologists, urologists, and nurses, is essential to ensure a complication-free procedure and successful ureteric stent removal. Finally, future research should focus on utilizing biodegradable and biocompatible materials to reduce and even eliminate the complications related to forgotten stents in order to reduce the financial burden associated with stent replacement and the management of stent-encrustation-related complications.

## 1. Introduction

Ureteral stents (or ureteral double-J stents [DJs]) play a pivotal role in urological practice [1]. These devices reduce obstruction and/or drain-infected upper urinary tract conditions when conservative treatments may fail, especially in critical conditions [2,3,4]. The use of this minimally invasive procedure has yielded an acceptable safety profile and is particularly beneficial in managing ureter hydronephrosis and urolithiasis in pregnant women [3,5].

During pregnancy, anatomical changes in the pyelocaliceal system, predominantly affecting the right side (due to anatomical relationships with iliac and ovarian vessels), lead to ureter hydronephrosis in over 40% of pregnancies [5]. This condition prevalently occurs during the third trimester [5]. Moreover, hormonal and immune changes in pregnant individuals increase the risk of infectious complications, threatening both maternal and fetal health [3,5,6,7]. The above modifications can also contribute to the formation of stones, further complicating the pregnancy and potentially leading to adverse outcomes such as preterm birth, preeclampsia, gestational hypertension, or maternal and fetal mortality [3,5,6,7].

Despite its benefits, ureteral stenting is not complication-free [8]. Up to 80% of patients may experience complications post-placement, although most are mild, such as discomfort or hematuria [8]. However, more severe complications, including urinary tract infections, stent encrustation, chronic obstruction, and subsequent loss of renal function, can occur [8,9]. Specifically, prolonged indwelling times increase the risk of stent encrustation, affecting 4–13% of stent patients [10,11]. Herein, we aimed to summarize available evidence of stent encrustation in pregnant women from physiopathological mechanisms to therapeutic options to improve their clinical and surgical management.

## 2. Materials and Methods

**Figure 1:**
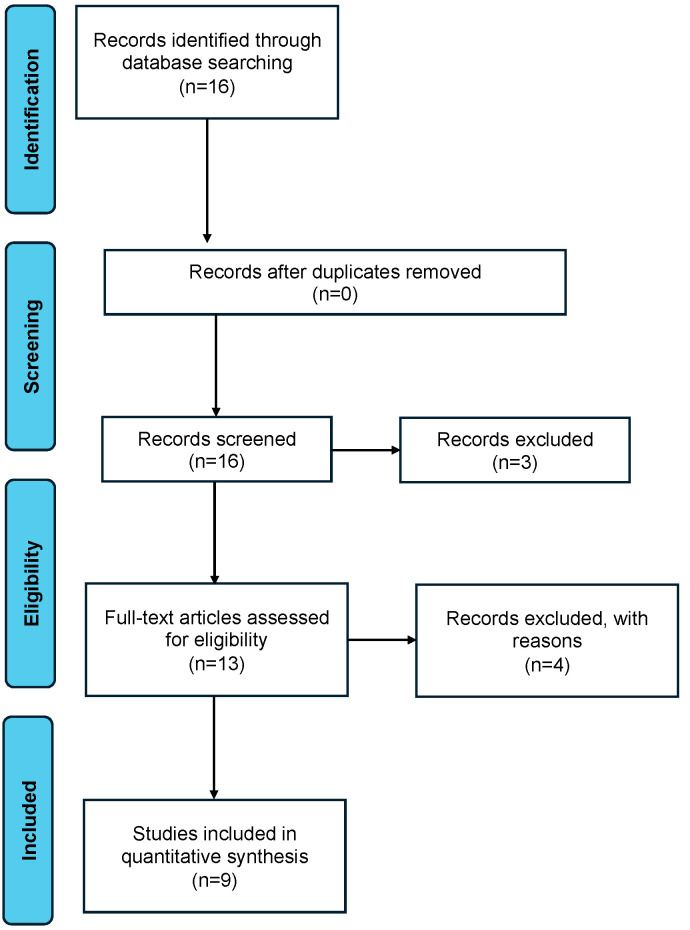
PRISMA statement.

We designed a narrative review of the studies investigating the rates, the causes, and the management options of stent encrustation during pregnancy. A comprehensive search in the MEDLINE, Scopus, and Cochrane Library databases was performed (Figure 1). Different combinations of the following keywords were used according to a free-text protocol: “stent encrustation”, “forgotten ureteral stent”, “calcified ureteral stent”, “encrusted ureteral stent “, “forgotten ureteric stent”, “calcified ureteric stent”, “encrusted ureteric stent” AND “pregnancy”. Articles in the English language published up to December 2023 with no chronological restriction were deemed eligible. Retrospective, prospective primary clinical studies, both comparative and non-comparative, meta-analyses, case series, case reports, and animal studies were included. Abstracts, conference abstract, editorials, and narrative reviews were excluded. The reference lists of the included papers were used to search other relevant articles. The selection of articles and data to be extracted was based on the authors’ expert opinions. Specifically, two urological senior residents in endourology and an aggregate professor in urology evaluated the papers collected. In the case of a discrepancy, a fourth investigator solved the disagreement. Reviewers were blinded to each other’s evaluations. In lieu of a formal ethics committee, the principles of the Helsinki Declaration were followed. According to the predefined study design, the results were qualitatively described, as reported in the primary studies, without quantitative synthesis (Table 1).

## 3. Physiopathological Mechanisms of Stent Encrustation

The mechanism of encrustation is complex and not yet known. We tabulated the most common causes (Table 2).

First and foremost, we take into account the systemic changes that occur during pregnancy [5]. For example, in the early stages of pregnancy, there is an increase in glomerular filtration and diuresis [5]. Additionally, elevated levels of circulating progesterone lead to reduced contractions of the ureteric smooth muscle, which can result in hydronephrosis, particularly affecting the right kidney [5]. Moreover, during the third trimester, the anteverted position of the uterus can exert extrinsic pressure on the ureter, further contributing to its dilation [5]. Additionally, the enlargement of the uterus may cause indentation of the bladder dome, resulting in the displacement of ureteral meatus to a higher position than usual, thereby predisposing to the formation of urinary stones [5].

Secondly, several authors recognized a pivotal role in stent encrustation due to urease-producing bacteria (such as Proteus, Pseudomonas, Klebsiella, and other Gram-negative pathogens) [20,21,22]. Specifically, these microorganisms were recognized as urinary tract infection causes in pregnant women with rates that ranged from 8.9% to 20% [20,21]. The bacteria contributed to the creation of colonies and biofilms. For instance, Barajas-Garcìa et al., in their case report, highlighted with high-quality images the architecture of crystalline biofilms found inside DJs from long-term stenting in renal transplant recipients [22]. The authors noticed that the external surface had a 3D structure, water channels, and patchy bacteria embedded in a high amount of extracellular matrix, consistent with previous findings [22,23,24]. Conversely, the lumen of the stent revealed bacteria biofilms in close connections through fimbriae-like structures [23,24]. A potential explanation may be represented by the low nutrient levels that may trigger extracellular matrix production on the external stent surface, while urine flow reduces this event in the lumen of the J stent. Furthermore, stent encrustation has also been observed under sterile conditions with the presence of calcium oxalate crystals [25]. It was assessed that calcium oxalate deposits were the main crystalline phase, mainly in the absence of urinary calcium phosphate, followed by calcium phosphate and ammonium magnesium phosphate [25]. During pregnancy, these sudden events are related to several metabolic prolithogenic factors that have intersected each other, such as hypercalciuria and hyperuricosuria [5,26,27]. As a result, urine flow constantly in contact with the DJ lumen plays a major role in the encrustation process [5,26]. Indeed, pH, the supersaturation of crystallizing substances, and a deficit of crystallizing inhibitors could create a structure gradually settling along the course of the stent, ending with obstruction [28,29,30].

Without a doubt, the indwelling time was assessed as the most-studied risk factor of stent encrustation [10,11,31,32]. Specifically, El-Faqih et al. were the first to observe how the indwelling time negatively influences stent encrustation, with a rate of 76.3% of the stents encrusted after 12 weeks since placement [31]. Then, Barajas-Garcìa et al. showed a similar rate of encrustation (almost 70%) after a median indwelling time of 60 days [22]. Subsequently, other authors, such as Kawahara et al. and Legrand et al., found comparable results in their studies [11,32]. Furthermore, DJ encrustations were recurrent in conditions in which the urinary bacterial load increases, such as recurrent urinary tract infections, diabetes mellitus, chronic kidney disease, and pregnancy [30,33]. Finally, Sighinolfi et al. studied the coating at both stent ends in 40 patients with DJs [30]. The authors identified that the proximal stent encrustation reflected the composition of stones in patients with urolithiasis [30]. Conversely, at the distal coil, urinary tract infections and patient aging influenced the levels of stent encrustation [32]. It should be noted that the prolonged indwelling stent time could increase the urinary tract infection occurrence rate, creating a vicious circle.

According to several authors, stent encrustation could be related to mechanical characteristic of the DJs [11,34]. For instance, Kawahara et al. assessed that the encrustation rates correlated with stent diameter rather than stent length or patency in 330 DJs in 181 patients retrospectively analyzed [11]. Moreover, the authors showed that the encrustation rate increased with the stent indwelling time (from 26.8% at less than 6 weeks to 75.9% at more than 12 weeks) [11]. Specifically, the 6 French stent developed more encrustation than the 7 F stent [11]. Conversely, Vogt studied the critical regions with abrupt changes in shape susceptible to stagnant flow and encrustation with a microfluidic-based model [35]. The author assessed that the alterations of the internal lumen of the stents may affect urine flow [35]. As a result, the low-velocity laminar vortices, present for instance in the cavity of a stent, promoted the deposition of encrustations and bacterial colonization [35].

## 4. Stent Encrustation Costs

The cost management of stent encrustation during pregnancy has no negligible financial burden due to the management of the event as well as the possible complications for both the mother and fetus. First and foremost, the higher the gestational age of a patient is, the more expensive the stone disease management costs will be [36,37]. Di Bello et al., in their clinical outlook, presented a discursive analysis of financial costs related to kidney stone disease during pregnancy [38]. Specifically, the authors enlightened that a complicated stent removal based on cystoscopy was two-fold more expensive than simple removal [38,39]. Additionally, encrusted stent removal costs on average 6.9 times (range 1.8–21 times) more than a standard stent removal [38,40]. As a result, considering the expenses per patient and assuming associated costs for pregnancy (in simple and in complicated scenarios), the financial burden of encrusted stent removal may result in 12-fold higher expenses for the healthcare system [38].

## 5. Clinical Presentation of Stent Encrustation

The clinical presentation of an encrusted stent in pregnant women is invariably heterogenous. Consistent with Ye et al. findings, urinary symptoms such as urgency, frequency, and dysuria were predominant symptoms of indwelling stents, coupled with micro-hematuria [19,41]. Other symptoms such as flank, urethral, and suprapubic pain and gross hematuria were minimal in contrast to other symptoms [5]. It should be noted that a forgotten and encrusted stent could determine recurrent urinary tract infections, with irritative symptom occurrence [25,42]. The magnitude of the symptoms ranged variably from different study cohorts. For instance, in Vajpeyi et al.’s report, irritative voiding symptoms (53%) were the most presented symptoms, followed by back loin symptoms (33%) [25]. Conversely, in Abdel-Kader’s study, renal colic was highly prevalent (73.9%), while fever and renal pain were recorded in 26.1% of cases [41]. Moreover, Thangavelou et al. showed the presence of the symptoms described above in 80% of 13 encrusted DJs before removal [26]. To sum up, Dai et al. concluded that the most common presenting symptoms of kidney stone disease and thus stent encrustation in pregnant women were flank pain, nausea/vomiting, micro-hematuria, and fever/chills [43]. These symptoms could influence the fetal growth and the homeostasis of the mother–fetus system [43]. Indeed, urolithiasis was historically associated with significantly higher adverse maternal outcomes, with a significant impact on fetal growth, such as preeclampsia, gestational hypertension, and diabetes [2,43,44]. Additionally, during pregnancy, kidney stone disease and stent encrustation can result in preterm birth [44]. Tangren demonstrated that in women without preexisting comorbidities (chronic kidney disease, hypertension, and diabetes), urolithiasis has a three times greater risk of preterm childbirth [45]. Consequently, a meta-analysis by Zhou et al. suggested speculatively that preterm birth may be associated with the overproduction of prostaglandin, promoting uterine contractions and fetal expulsion, due to vomiting and dehydration [44]. Finally, kidney stones were associated with an increased risk of urinary tract infections, identified previously as a preterm birth triggers [4,44].

## 6. Imaging for Stent Encrustation Diagnosis

### 6.1. Techniques

In the general population, kidney–ureter–bladder (KUB) radiography, non-contrast computed tomography (NCCT), and KUB ultrasonography are commonly used for imaging evaluation [46]. These techniques may confirm the diagnosis, defining the location of encrustation (single or multiple), the consequent extension, and the grading of severity [46]. The advantages of those imaging investigations outstand radiographs alone, which often do not allow mapping the site of calcification accurately [47]. As recently noticed in Wang et al.’s report, relying on 147 studies with 1292 patients included, the rates of use of KUB radiography, NCCT, and KUB ultrasonography techniques are 96.8%, 76.1%, and 45.2%, respectively [46]. However, during pregnancy, the protocols that urologists usually adopt in the management of stent encrustation cases are undermined. Indeed, to protect the mother–fetus system, the exposure to radiation is contraindicated due to high teratogenesis and carcinogenesis risks [9]. Specifically, the highest effects on fetuses occurred during the 2nd to 15th week of gestation [48]. Thus, both KUB and CT scans should be avoided unless necessary. According to international guidelines, ultrasounds are recommended as the first-line investigation [1]. Ultrasonography can show hydronephrosis, dilatation of the proximal ureter, and the presence of fragments and calcified stents both in the renal pelvis and in the bladder [3,19,47,49,50]. Doppler ultrasonography can be used to measure the renal resistive index (RI) (defined as [peak systolic velocity − end diastolic velocity]/peak systolic velocity], which can distinguish gestational ureter hydronephrosis from obstructions [51,52]. Moreover, color-flow Doppler ultrasonography can reveal the presence of a twinkling artifact represented by the encrustation of DJs [13]. Finally, transvaginal ultrasonography can add to abdominal ultrasonography to identify the distal urinary tract [53]. Taken together, those techniques can allow clinicians to grade the severity of encrustation, providing a tangible tool to predict intra- and post-operative complications [47,54,55].

### 6.2. Grading of Severity of Stent Encrustation: FECal

According to Acosta-Miranda et al.’s approach, a grading system of encrustation was developed in 2009 [56]. The FECal (forgotten, encrusted, and calcified) DJ classification consisted of five levels of stone burden:

- Grade I: Minimal linear inlay on any of the jacks.
- Grade II: Circular inlay enclosing any of the jacks.
- Grade III: Circular incrustation that completely encloses any of the sides, with linear portions of incrustation in the ureteral portion of the catheter.
- Grade IV: Circular inlay that completely encloses both jacks.
- Grade V: Diffuse and protruding inlay that completely encloses both jacks and the entire ureteral portion.

### 6.3. Grading of Severity of Stent Encrustation: KUB

The kidney–ureter–bladder (KUB) system proposed by Arenas et al. is the sum of the stone burden scores of three different parts of an encrusted stent [57]. Specifically, within the kidney, ureter, and bladder, for each location a score is determined using a scale from 1 to 5, according to the maximal diameter of encrustation. The score for each location is obtained considering the encrustation of the coil (renal, ureteral, and/or bladder), the maximal width (≤5 mm), and the extension (≤5 mm). Higher scores are related to a higher burden of stone disease after the procedure.

### 6.4. Grading of Severity of Stent Encrustation: V-GUES

The Visual Grading for Ureteral Encrusted Stent (V-GUES) tool has been recently developed by Manzo et al. [58]. The tool may aid urologists in determining the likelihood of treatment success of removing both the stent and remaining stone fragments. The severity of stent encrustation is graded from “A” (lowest) to “D” (the highest).

### 6.5. Practical Considerations

Despite the tools that urologists own to better understand the location and the severity of stent encrustations, pregnancy remains a challenging scenario. First, KUB ultrasonography is the only tool reliable in a non-emergency setting during the pregnancy period due to its non-invasiveness and safeness. Despite these considerations, KUB ultrasonography does not allow as detailed a definition of the grading system as its X-ray-based technique counterparts, undermining the decision of the optimal strategy to treat stent encrustation in pregnant women. Second, the tools for predicting the success rate and to plan the treatment strategies are based on X-ray evaluation. As a result, those validated tools may be inapplicable to pregnancies. Taken together, this represents an unmet need. It is urgent to develop a tool feasible and actionable during pregnancy to help clinicians and urologists to promptly evaluate and manage the condition of stent encrustation that, albeit rare, has its own morbidity.

## 7. Management of Stent Encrustation during Pregnancy

### Operative Management

Management for pregnant patients is influenced by several factors, including their pre-operative condition, the severity and grading of encrustations, as well as the presence, size, and location of stones. It is important to note that stent migration and/or fragmentation may occur during the procedure, potentially altering the intervention plan [25]. Undoubtedly, in the presence of stent encrustation, every endoscopic manipulation of forgotten stents should first and always be preceded by appropriate imaging to decide the safest removal strategy both for the mother and for the fetus [47,49]. Second, force should be avoided if removal of the stent cannot be managed by a simple cystoscope to reduce ureteric injury occurrence [49]. Generally, a multimodal approach is necessary for moderate-to-severe fouling, particularly when a single treatment modality fails. Historically, ureterorenoscopic lithotripsy with stent removal (URS) represented the surgical choice to manage stone disease and encrusted stents in pregnant patients [49]. Specifically, it can be performed under local anesthesia plus sedation and is feasible in all trimesters of gestation [50,59]. Interestingly, a comparative study relying on 38 pregnant women who underwent percutaneous nephrostomy (PCN) versus 46 pregnant women who underwent URS, showed the feasibility of PCN after the third trimester for the management of HUN [60]. When the anesthesiology risk is non-negligible, indeed, PCN is more effective and feasible than ureteric stent insertion as a treatment for symptomatic HUN during pregnancy due to its reduced risk of reintervention [60]. The main factor that predicts either the necessity of multiple surgeries or associated complications is the proximal stone burden. The lower encrusted segment in the bladder and in the ureteral part is always released first before dealing with the surgical removal of the proximal end part. New modalities to remove encrusted stents in the general population as well as in pregnant women have emerged in recent years. Indeed, according to Thangavelu et al. in their multicentric study on 13 encrusted stents in twelve patients, the first step of encrusted DJ removal was cutting the stent with a Holmium LASER during URS to create space for ureteral access [3,26]. Specifically, Watterson et al. showed that the coupled use of a Holmium LASER and pneumatic lithotripsy may also be useful during pregnancy [14]. Specifically, a Holmium LASER applied through semirigid ureteroscopes may deliver the energy to a very localized area with minimal or no collateral damage [14]. The authors reported no complications among the newborns, in general, or in terms of deafness [14]. Thus, the Holmium LASER achieves the feasibility and tolerability needed to be adopted in the current stent encrustation management of pregnant women. However, this technique is not available in all the centers, and it should be performed by experts in the field. It is mandatory to mention the role of external shock wave lithotripsy (ESWL), which is specifically and largely used in the management of non-pregnant subjects [33,61,62]. This procedure during pregnancy is difficult and dangerous to perform both for the mother and for the fetus. Specifically, the slow emission of fragments after ESWL as well as the need to add a second endoscopic (or surgical) step for DJ removal has resulted in inherent difficulties during pregnancy [33,63,64]. However, it should be acknowledged that Asgari et al. inadvertently treated six pregnant women with ESWL for renal stones during their first month of pregnancy. Fortunately, the procedure did not result in fetal effects. However, ESWL may increase the risk of premature delivery and fetal death, and it should avoided [41,61,65]. In conclusion, a step-by-step approach should be adopted in pregnant women [63,64]. First, a DJ encrustation classification should be performed [63,64]. Second, a calculation of the stone load should be carried out, and finally, the identification of the encrustation side should be completed [63,64]. After the evaluation of the above steps, a multidisciplinary team headed by an experienced endourologist with high-volume experience, following thorough consultation with obstetricians, gynecologists, radiologists, and anesthetists, should evaluate the possible options for the patient. URS should always be considered as the first option, as well as PCN when URS is not applicable. In well-trained hands, Holmium LASER lithotripsy, due to its safety in all stages of pregnancy, may also be performed [14]. However, it should be acknowledged that to complete the encrusted stent removal, if previous procedures fail, retrograde intrarenal surgery (RIRS) may be performed to deal with proximal coil encrustation in highly selective cases [14,65].

## 8. Preventive Management and Future Perspective

DJ materials have evolved substantially to reduce the encrustation phenomenon. An indwelling time longer than 4–6 weeks [66] represents one of the predisposing factors for stent encrustation, and as a consequence, bacterial colonization plays a crucial factor in the stent encrustation process. Ch’ng et al. investigated the relationship between the use of sodium citrate and stent encrustation in 115 patients who underwent lithotripsy surgery [29]. They found no statistical differences between the encrustation group and the control group (52.6% vs. 46.6%) [29]. Conversely, Mohammadi et al. studied, in a randomized-clinical trial of 65 patients, the effect of potassium citrate in stent encrustation [67]. Their analysis revealed that potassium citrate after DJ placement significantly decreases the formation of calcium oxalate and uric acid encrusted material on DJs [67]. Thus, it may represent an alternative option in the conservative and preventive management of stent encrustation during pregnancy [67]. Thus, research has focused on designing materials or layering that reduces this activity. Most stents now being used consist of polyurethane or copolymers that reduce bioactive substance adhesion and aggregation. Specifically, the stent surface could influence the dissociation of catabolite determination in stent encrustation. For instance, Yao et al. improved the stability of the coating on polymeric stents using Cu^2+^-coordinated dopamine self-polymerization. Then, a cysteine-terminated antimicrobial polymer (AMP) was introduced on the main coating [68,69]. The authors showed that both in vivo and in vitro, the stent had improved stability and inhibited bacterial growth and biofilm formation with an acceptable biocompatibility [68,69]. Interestingly, recent studies demonstrated the role of N-Acetylcysteine (NAC) in the management of recurrent UTIs [70,71]. Specifically, NAC has exhibited urease activity suppression at low concentrations, as well as bacteriostatic and antibiofilm activities, resulting in the prevention of catheter occlusion [70,71]. As a consequence, the reduction in biofilm formation may also impact the urinary tract microbiota, reducing the organic matrix that contributes to the encrustation mechanism [70,71]. Future research could test this evidence in a wider group and in clinical trials. Recently, the concept of biodegradability has emerged. Wang et al. studied the role of biodegradable poly-L-lactic acid (PLLA) in the management of ureteral stricture in vivo and in vitro [72]. The results were encouraging, depicting a novel scenario that could revolutionize the management of stent placement [72]. Indeed, the mesh DJs proposed by Wang had a reliable ability in supporting the ureter, solving ureter obstruction [72]. Additionally, the rate of urinary tract infections and stent encrustation was lower in cases with DJs [72]. Moreover, its indwelling time was from 3 to 5 months since the implantation, without the need for a second surgery for stent removal [72]. These results could prospectively reduce both the complication of forgotten stents and the financial burden of replacing the stents, representing a fundamental choice in the management of delicate conditions, such as pregnancy. Virtually, pregnant women candidates to receive ureteric stent placement should be pre-operatively counseled about the risk of complication of an indwelling stent longer than 4–6 weeks [66]. Moreover, they should be routinely screened in order to avoid the “forgotten stent” phenomenon as well as the occurrence of stent encrustation. Ideally, as previously observed, the ureteric stent should be removed within the first four to six weeks from its placement [66]. Lifestyle changes and UTI prevention should also be encouraged. On the other hand, research should be promoted to build new, feasible devices. Moreover, clinicians should be granted to use these new devices, reducing the economic burden of ureteric encrusted stent removal.

## 9. Strengths and Limitations

To the best of our knowledge, this is the most up-to-date review on this topic. We described the most current evidence on stent encrustation during pregnancy, highlighting its clinical presentation, its costs, and its potential complications for the fetus and for the mother in pregnant women with encrusted stents. However, this paper should be read considering several limitations. The main weakness is the narrative design and the consequent absence of quantitative synthesis of the data (meta-analysis). However, the paucity, heterogeneity, and low-to-intermediate quality of the available data may have made the data synthesis unreliable.

## 10. Conclusions

Ureteric stents can properly manage both ureter hydronephrosis and urolithiasis during pregnancy. Stent encrustation is caused by multifactorial causes such as urinary tract infection occurrence and recurrence, prolonged indwelling time, and biochemical urinary characteristics. A step-by-step as well as multimodal approach is required to guarantee a complication-free procedure and the efficacy of the encrusted stent removal. First, DJ encrustation classification should be performed. Second, a calculation of the stone load should be undertaken, and finally, the identification of the encrustation side should be completed. After the evaluation of the above steps, endourologists, along with other physicians (obstetricians, gynecologists, radiologists, and anesthetists), should evaluate the possible options for the patient. Ureteroscopy should be always considered as the first option. Future research should implement the use of biodegradable and biocompatible materials to reduce both the complication of forgotten stents and the financial burden of managing stent-replacement- and stent-encrustation-related complications.

## Figures and Tables

**Table 1:** Study characteristics of nine studies on stent encrustation during pregnancy included in the qualitative evaluation analyses.

	Number of Pregnant Women hh *	Type of Study	Imaging Technique for Stent Encrustation Diagnosis	Cost Analysis	Complications
Borboroglu 2000 [12]	3	Single institution Case series	N.A.	N.A.	Sepsis
Trillaud 2001 [13]	2	Case report	Color doppler US	N.A.	-
Watterson 2002 [14]	2	Multi-institutional Retrospective study	Trans-abdominal US X-ray technique	N.A.	-
Khan 2012 [15]	1	Case Report	US	N.A.	Pyrexial UHN progression Emergency nephrostomy
Ngai 2013 [16]	3	Single institution Retrospective study	Trans-abdominal US	N.A.	-
Pais 2014 [17]	2	Single institution Retrospective study	KUB radiograph CT scan	N.A.	-
Thomas 2017 [18]	4	Single institution Retrospective study	N.A.	N.A.	Non-obstructive pyelonephritis
Ye et al., 2021 [19]	1	Case report	Trans-abdominal US	N.A.	-
Radu 2022 [4]	2	Single institution Retrospective study	US MRI	N.A.	-

* Number of the pregnant women with encrusted ureteric stent. Abbreviations: UHN, hydroureteronephrosis; LUTS, lower urinary tract symptoms; MRI, magnetic resonance imaging; N.A., not available; KUB, kidney ureteral bladder; SE, stent encrustation; US, ultrasound sonography.

**Table 2:** Common causes of stent encrustation in pregnant women, divided into pregnancy-related, environmental-related, and ureteric-stent-related causes.

**Pregnancy Related**	
Physiological changes	Hormonal changes (progesterone increasing)
	Glomerular filtration increasing
	Hypercalciuria
	Hyperuricosuria
Anatomical changes	Uterus in anteversion position
	Bladder dome indentation due to uterus enlargement
**Environmental related**	
Pathogen colonization	Urease-producing bacteria
Urine characteristics	Calcium oxalate crystal production
	Deficit of crystallizing inhibitors
	pH alterations
**Ureteric stent related**	
Ureteric stent	Materials
	Stent diameter
	Indwelling time

## Data Availability

Not applicable.
